# Target-Site Mutations Conferring Herbicide Resistance

**DOI:** 10.3390/plants8100382

**Published:** 2019-09-28

**Authors:** Brent P. Murphy, Patrick J. Tranel

**Affiliations:** Department of Crop Sciences, University of Illinois, Urbana, IL 61801, USA; brentpm2@illinois.edu

**Keywords:** D1 protein, acetolactate synthase, tubulin, ACCase, EPSPS, phytoene desaturase, PPO, glutamine synthetase, auxin

## Abstract

Mutations conferring evolved herbicide resistance in weeds are known in nine different herbicide sites of action. This review summarizes recently reported resistance-conferring mutations for each of these nine target sites. One emerging trend is an increase in reports of multiple mutations, including multiple amino acid changes at the glyphosate target site, as well as mutations involving two nucleotide changes at a single amino acid codon. Standard reference sequences are suggested for target sites for which standards do not already exist. We also discuss experimental approaches for investigating cross-resistance patterns and for investigating fitness costs of specific target-site mutations.

## 1. Introduction

Herbicide-resistance mechanisms broadly fall under two categories: target-site mechanisms and non-target-site mechanisms [1,2]. The former involves a change to the molecular target of the herbicide (usually an enzyme) that decreases its affinity for the herbicide. Although much less common, target-site resistance can also occur via increased expression of the target, which results in more herbicide required to achieve a lethal effect [3,4]. Non-target-site resistance encompasses any mechanism that reduces the amount of herbicide that reaches the target site, or that ameliorates the effect of the herbicide despite its inhibition of the target site.

Our understanding of specific DNA changes that confer non-target-site resistance is still in its infancy [5]. In contrast, the first DNA change conferring evolved target-site resistance (to triazines) was identified over three and a half decades ago [6]. Since then, numerous resistance-conferring mutations have been identified from dozens of weed species and now span nine herbicide target sites (Table 1). The purpose of this review is to provide an update of new mutations that have been recently identified for each of these nine target sites. In this review, we consider “new mutations” to be those that confer an amino acid change that has not been reported previously from any weed species. Notably, gene duplication is not within the scope of this review. While gene duplication events have been observed to confer resistance to herbicide Groups 1 and 9 [3,4], the underlying genetic mechanisms for resistance evolution lie outside of the applicable target-site coding region. We discuss each target site sequentially, beginning with a brief description of the target. We then highlight the most recent reviews of mutations for each target before reviewing new mutations associated with each target. Resistance cases are discussed in order of WSSA group number, with the HRAC classification listed in parenthesis [7]. 

## 2. Summary by Herbicide Group

### 2.1. Acetyl-CoA Carboxylase Inhibitors: Group 1 (A)

The basis of function for Group 1 chemistries was reviewed in detail by [16], and target-site resistance to Group 1 chemistries was last reviewed in 2014 [17]. Briefly, acetyl-CoA carboxylase (ACCase) catalyzes the carboxylation of acetyl-CoA to malonyl-CoA through a two-step, reversible reaction. First, biotin in complex with the enzyme is carboxylated, and second, the carboxyl group of biotin is transferred to acetyl-CoA, producing malonyl-CoA [16]. ACCase is composed of three domains: the biotin carboxylase domain, biotin carboxyl carrier protein domain, and the carboxyltransferase domain. Inhibitors of ACCase are classified within three chemical families: the aryloxyphenoxypropionates (FOPs), cyclohexanediones (DIMs), and phenylpyrazolin (DEN) [17]. Although ACCase is present within the cytoplasm and chloroplast, ACCase inhibitors affect only the homomeric, plastidic ACCase isoform specific to the Poaceae family, through nearly competitive, reversible inhibition [17]. By convention, amino acid numbering follows the *Alopecurus myosuroides* sequence, CAC84161 [17]. The crystal structures of the ACCase carboxyltransferase domain, as derived from yeast, have been produced both in a ‘native’ state [18] and in complex with representative chemicals from all three herbicide families within the group [19,20,21]. 

Target-site ACCase resistance mutations commonly evolve in grass weed species in response to selection and show great diversity in terms of mutation sites. Codon changes at positions 1781, 1999, 2027, 2041, 2078, 2088, and 2096 have been previously reviewed [17]. Subsequently, two new substitutions have been reported in weedy species, including one at a new site. 

The Ile-2041 position has been well characterized for resistance to FOP chemistries. In China, a new substitution, Ile-2041-Thr, was observed in *Alopecurus aequalis* [22]. Dose–responses conducted on segregants of a single, heterozygous plant revealed resistance to a number of FOP chemistries and sensitivity to several DIMs. In addition, reduced sensitivity towards pinoxaden, a DEN chemistry, was reported. Reduced sensitivity, and even resistance, caused by substitutions at the Ile-2041 position have been observed to pinoxaden, although this was inconsistent between species. For instance, the Ile-2041-Asn substitution has been reported to cause a high level of resistance to pinoxaden in *Beckmannia syzigachne* [23], while providing reduced sensitivity in *Lolium multiflorum* [24]. Functional validation is required to reach a consensus on the effects of Ile-2041 position on DEN chemistries.

First identified in *Eleusine indica* from Malaysia, the Asn-2097-Asp substitution is suggested to provide resistance to fluazifop, a FOP [25]. While the functional effects of substitutions at the Asn-2097 position are uncharacterized, substitutions in the proximal Gly-2096 position have been characterized to confer FOP-specific resistance [26]. However, the contribution of this new substitution to the resistance was not explored further. As heterozygous individuals within the *E. indica* population of interest were identified, cosegregation studies would provide greater evidence of the importance of Asn-2097 towards Group 1 inhibitor resistance.

### 2.2. Acetolactate Synthase Inhibitors: Group 2 (B)

Resistance to acetolactate synthase (ALS) inhibitors was most recently reviewed in 2014 [27]. Acetolactate synthase, also referred to as acetohydroxyacid synthase (AHAS), is a dual-functioning enzyme. The enzyme catalyzes both the synthesis of acetolactate from two pyruvate molecules, and the synthesis of acetohydroxybutyrate from ketobutyrate and pyruvate, where both reactions require thiamin diphosphate, FAD, and Mg^2+^ [28]. ALS is the first enzyme in the synthesis pathway for the branched-chain amino acids valine, leucine, and isoleucine, and the depletion of these amino acids is the mode of action for ALS-inhibiting herbicides [27]. Encoded in the nucleus, the enzyme is localized to plastids. Group 2 is composed of five herbicide families: sulfonylurea (SU), imidazolinone (IMI), triazolopyrimidine (TP), pyrimidinyl-thiobenzoate (PTB), and sulfonyl-aminocarbonyl-triazolinone (SCT). Crystal structures of *Arabidopsis thaliana* in complex with SU, IMI, TP, PTB, and SCT have been generated [29,30,31]. The naming convention for amino acid substitutions is based on the *A. thaliana* sequence AY124092, and is in agreement with the available crystal structures. 

The relative ease at which weeds evolve resistance to ALS inhibitors is the Achilles heel of these herbicides, and resistance is often due to target-site substitutions [32]. Evolved resistance in weeds has been attributed to substitutions at each of the following eight different amino acids: Ala-122, Pro-197, Ala-205, Asp-376, Arg-377, Trp-574, Ser-653, and Gly-654. Often, several different substitutions at each of these eight sites are able to confer resistance. Since the last review, six new substitutions (at previously reported sites) have been reported in weedy species and are summarized herein. 

The Ala-122-Asn substitution was first reported in *Echinochloa crus-galli* from Italy [33]. This substitution has been reported to confer resistance to SU chemistries in yeast [34], and substitutions at this position can result in resistance to both SU and IMI chemistries [27]. Resistant and sensitive plants from the same field location were identified and bulked, and resistance was characterized to be representative of SU, IMI, TP, and PTB chemistries. Sequence analysis was used to identify the amino acid substitution, and in vitro ALS enzyme activity bioassays supported observed resistance, indicative of a target-site resistance mechanism. A fitness cost under ideal growth conditions, but not under competition, was identified through comparative growth analysis. 

The Ala-122-Ser substitution was first reported in *Amaranthus palmeri* from Argentina [35]. Previously, the Ala-122-Ser mutation has been reported to confer resistance to SU chemistries in yeast [34]. Greenhouse screening identified consistent resistance to representative SU, IMI, and TP chemistries when compared to an unrelated sensitive population. In vitro ALS enzyme activity bioassays support resistance to the representative SU and IMI chemistry, but not to the representative TP chemistry. Sequence analysis on a set of eight resistant plants revealed that the population was a composite between the Ala-122-Ser, Ser-653-Asn, and several uncharacterized substitutions (Pro-84-His and Ala-282-Asp). The Ala-122-Ser mutation was observed only in combination with the uncharacterized Ala-282-Asp substitution. Functional characterization in the absence of other substitutions is necessary to confirm this potential resistance mechanism. 

As first published in 2015 by Liu et al. [36] in *Myosoton aquaticum* from China, the Pro-197-Glu substitution confers resistance to representative SU, IMI, TP and PTB chemistries. Substitutions at the Pro-197 site are generally considered to be SU-specific [27]. A homozygous-resistant population was developed through genotypic selection, and dose–response compared to an unrelated sensitive population identified uniform resistance. In vitro ALS enzyme activity bioassays confirmed reduced sensitivity of the ALS enzyme when compared to the sensitive population for the SU, IMI, TP, and PTB chemical families. The response of the Pro-197-Glu substitution to the SCT chemistries is unknown. 

The Pro-197-Phe substitution was first reported in *Sisymbrium orientale* in Southern Australia [37]. A population survey of the species was conducted for resistance to SU and IMI chemistries, where sequence analysis of survivors was conducted. Of the 65 populations under investigation, one population was reported to consistently possess the Pro-197-Phe substitution, the result of a double nucleotide substitution when compared to the wild-type sequence. This population was resistant to a representative SU chemistry, while sensitive to an IMI chemistry. To date, functional validation of the Pro-197-Phe has not been conducted. The effect of the Pro-197-Phe substitution on the TP, PTB, and SCT chemical families is unknown.

The Ala-205-Phe substitution was first reported in *Poa annua* in Tennessee, US [38]. Substitutions at the Ala-205 position are considered to confer SU-specific resistance [27]. Dose–response with representative SU chemistry [39], and delimiting rate of a representative IMI chemistry, identified in planta resistance when compared to an unrelated sensitive population. Sequence analysis revealed the Ala-205-Phe substitution, as derived from a double nucleotide substitution. These substitutions were artificially introduced to the *A. thaliana* gene sequence, expressed, and purified from *Escherichia coli* for use in in vitro ALS enzyme activity assays. These in vitro assays revealed resistance responses when compared to wild-type to a wide range of chemistries from each of the SU, IMI, TP, PTB, and SCT chemical families. Interestingly, no resistance was observed to florasulam, though this observation was not confirmed in planta, perhaps due to the lack of efficacy of florasulam on grasses. The in planta response of the Ala-205-Phe substitution to the TP, PTB, and SCT chemical families is unknown. 

The Trp-574-Arg substitution was first reported in *Digitaria sanguinalis* in China [40]. Substitutions at the Trp-574 site have been documented to confer broad cross-resistance to the ALS-inhibiting chemistries. Dose–response revealed resistance to representative SU, IMI, and TP chemistries when compared to an unrelated sensitive population. Reduced sensitivity of the ALS enzyme to the SU, IMI, and TP chemistries was characterized with in vitro ALS enzyme activity assays. The response of the Trp-574-Arg substitution to the PTB and SCT chemical families is unknown.

### 2.3. Microtubule Inhibitors: Group 3 (K_1_)

Resistance to microtubule inhibitors was last reviewed in 2010, though the dinitroaniline trifluralin was reviewed in 2013 [1,41]. Tubulin heterodimers, composed of ɑ- and ꞵ-tubulin, polymerize to form microtubules. Microtubules are key structural polymers, which mediate multiple cellular processes through the dynamic reorganization of microtubules [42]. Microtubule inhibitors bind to the ɑ-tubulin subunit in a reversible, competitive manner, deregulating the organization of microtubules [41,42,43]. Currently, there are five classes of microtubule inhibitors: dinitroaniline, phosphoroamidate, pyridine, benzamide, and benzoic acid. The crystal structure of bovine tubulin heterodimer is available, which has been used to computationally determine dinitroaniline binding domains and validated through mutagenesis studies [42,44,45]. Because ɑ-tubulin is highly conserved, the need for a reference sequence is minimal. If necessary, the authors recommend the *Setaria viridis* sequence CAE52514. 

Resistance to Group 3 herbicides is relatively rare (reported in just 12 weed species). As of 2013, ɑ-tubulin substitutions implicated in resistance included Leu-125-Met, Leu-136-Phe, Val-202-Phe, Thr-239-Ile, and Met-268-Thr [1,41]. Target-site resistance to microtubule inhibitors is unique among herbicides in that it is a recessive trait. This recessive nature likely accounts in part for the relative rarity of target-site resistance to these herbicides (especially, in out-crossed species) [1], despite the fact that there appear to be numerous substitutions that can confer resistance. 

The substitution of Arg-243 to Met or Lys was first reported in *Lolium rigidum* from Western Australia [46]. Dose–responses of multiple dinitroaniline compounds revealed resistance when compared to an unrelated sensitive population [47]. Sequence analysis revealed a subset of the population possessed Arg-243 substitutions, though in combination with the known substitutions Thr-239-Ile or Val-202-Phe. Transgenic ɑ-tubulin genes carrying wild-type (Arg-243), Arg-243-Met, and Arg-243-Lys were introduced into *Oryza sativa* and resistance to multiple dinitroaniline chemistries in calli lines with similar recombinant protein abundance levels were observed for the mutant but not the wild-type genes. Structural modelling revealed the substitutions are expected to reduce binding efficiency between trifluralin and the tubulin subunit. The interaction of these Arg-243 substitutions with the other substitutions were not documented. 

### 2.4. Synthetic Auxins: Group 4 (O)

Herbicides within Group 4, the synthetic auxins, are synthetic analogues of the endogenous plant hormone indole-3-acetic acid (IAA) [48]. The deregulation of auxin-dependent plant signalling pathways results in the efficacy of these herbicides for weed control. Synthetic auxin chemistries are largely preferential towards dicots, with the exception of quinclorac, which has grass activity. A more comprehensive review of the mode of action of synthetic auxin chemistries is provided by Grossmann [49]. Resistance to synthetic auxin chemistries was last reviewed in 2018, though only non-target-site resistance mechanisms had been published in weedy species at that time [48]. Currently, synthetic auxins are separated into seven classes: phenoxy-carboxylates, quinolone-carboxylates, pyrimidine-carboxylates, benzoates, pyridine-carboxylates, pyridyloxy-carboxylates, and arylpicolinates [48]. Unlike other herbicide groups, which target a specific protein, synthetic auxins interact with numerous proteins, including from the following families: TIR1 and Auxin F-Box [50], AUX/IAA protein, AUX1/LAX influx carrier, PIN efflux carrier, and ABCB [51]. To date, however, evolved resistance to synthetic auxins has only been reported in the AFB and AUX/IAA families, and as such, these are discussed here.

In *A. thaliana*, there are six members of the AFB family, TIR1 and AFB1-5 [52]. All members of the AFB family are nuclear-encoded and localize to the nucleus [52]. Selective, or perhaps preferencial, binding of synthetic auxin chemistries to subsets of these protein targets has been reported [53], which suggests the loss of sensitivity of a few, or even one, of these receptors may result in herbicide resistance. In *A. thaliana*, there are 29 AUX/IAA genes [54]. AUX/IAA proteins are co-receptors, which pair with AFB proteins in the presence of auxin to form a larger co-receptor complex, termed the SCF^TIR1 [55]. The crystal structure of the TIR1-ASK1 has been developed in the native state and in complex with IAA, 1-naphthalene acetic acid (1-NAA), and 2,4-dichlorophenoxyacetic acid (2,4-D) [56]. As, prior to 2018, target-site resistance to synthetic auxins had not been reported, no convention has been established for amino acid numbering.

In 2018, LeClere et al. [15] were the first to identify a target-site resistance mechanism in *Kochia scoparia* to representatives of three classes of synthetic auxins: benzoates, phenoxy-carboxylates, and pyridine-carboxylates. Root length assays and dose–response analysis identified resistance to the aforementioned chemical classes. Transcriptome sequencing and sequence analysis identified a two-nucleotide substitution that results in a single amino acid substitution in the highly conserved GWPPV/I region (GWPPV/I→NWPPV/I) of KsIAA16. Yeast Two-Hybrid assays demonstrated a loss of interaction between the KsIAA16 and KsTIR1 in the presence of representative benzoates, phenoxy-carboxylates, and pyridine-carboxylates as a result of the Gly-73-Asn substitution (as numbered based on the *A. thaliana* IAA16 sequence). Co-segregation analysis in the F2 generation suggests benzoate resistance is linked to the observed nucleotide substitutions. Resistance mediated through the Gly-73-Asn substitution was confirmed through complementation in *A. thaliana*. A fitness cost associated with the Gly-73-Asn substitution was observed within F2 segregants. 

As LeClere et al. [15] were the first to document target-site resistance to synthetic auxins, the amino acid numbering convention should follow their example. Within their research, LeClere et al. [15] utilized the *A. thaliana* sequence AT3G04730 to number amino acids. If resistance-conferring mutations are found on other auxin receptors, we propose amino acid numbering should occur, following the most appropriate gene within *A. thaliana*.

### 2.5. Photosystem II Inhibitors: Groups 5 (C_1_), 6 (C_3_) and 7 (C_2_)

Herbicide resistance to photosystem II inhibitors was last reviewed in 2010 [1]. Photosystem II inhibitors, while spread over three herbicide groups, are reversible, competitive inhibitors of the Qb-binding niche of the D1 protein in the photosystem II complex. Photosystem II inhibitors are further subclassified into numerous chemical classes [57]. Group 5 contains the phenyl-carbamates, pyridazinones, triazines, triazinones, triazolinones, and uracils. Group 6 contains the benzothiadiazinones, nitriles, and phenyl-pyridazines. Group 7 contains the amides and ureas. Inhibition results in a disruption of the chloroplastic electron transport chain, resulting in the build-up of reactive oxygen species. The D1 protein is encoded by the chloroplastic psbA gene, and is expressed within the chloroplast. The crystal structure of the D1 protein was derived from the L-protein of *Chlamydomonas reinhardtii* [58], and more recently, from other purple bacteria [59]. Amino acid numbering is based on *A. thaliana*. 

As mentioned in the Introduction, the first identified resistance-conferring target-site mutation was to the photosystem II inhibitors. This mutation caused a Ser-264-Gly change and, since, has emerged as the dominant mutation for resistance to these herbicides. Other mutations, known as of 2010, included Val-219-Ile, Ala-251-Val, Phe-255-Ile, Ser-264-Thr, and Asn-266-Thr. Target-site resistance to photosystem II inhibitors is unique in that, because the target site is encoded by a plastidic gene, resistance is expected to be maternally inherited in most, if not all, weed species. 

A Leu-218-Val substitution was first reported in *Chenopodium album* from Germany [60]. Dose–response identified resistance to triazinone, but not triazine chemistries. Sequence analysis identified the Leu-218-Val substitution unique to the resistant biotype. While the Leu-218 amino acid is part of the Qb-binding niche, modelling was not conducted to determine the impact of the observed substitution. Although Leu-218-Val has not been functionally validated, it is adjacent to the Val-219 site, where substitutions have been documented to confer resistance to Groups 5 and 7 [61]. Inheritance of the observed resistance was not conducted, but it is expected to be maternally inherited. 

A Phe-274-Val substitution was reported in *Raphanus raphanistrum* from Western Australia [62]. This is the first report of a resistance-conferring substitution at or near the Phe-274 site. Delimiting dose and dose–response analysis identified a resistant response to representative Group 5 and 7 chemistries, but increased sensitivity to a representative Group 6 chemistry. Structural modelling suggests that the Phe-274-Val substitution results in a weakening in binding efficiency of representative Group 5 and 7 chemistries. Inheritance of the observed resistance was not conducted and the substitution has not been functionally validated.

### 2.6. EPSP Synthase Inhibitors: Group 9 (G)

5-enolpyruvylshikimate-3-phosphate (EPSP) synthase catalyzes the reaction between phosphoenol pyruvate and shikimate-3-phosphate to produce EPSP and inorganic phosphate [63]. Resistance to EPSP synthase inhibitors has been well reviewed in 2014 [64] and 2018 [65]. EPSP synthase is a nuclear-encoded, chloroplast-localized enzyme required for aromatic amino acid production [66]. Glyphosate, the only chemistry within Group 9, is a slowly reversible to irreversible competitive inhibitor at the phosphoenol pyruvate binding pocket [63]. The crystal structure of EPSP synthase, as derived from *E. coli* in complex with glyphosate, is known [63]. The numbering of amino acids is referenced against the start of the mature EPSP synthase enzyme of plants, such as the *A. thaliana* sequence, AT2G45300 [64]. 

Target-site resistance to Group 9 is unique due to the apparent necessity for multiple amino acid substitutions to confer a strong phenotypic response. Substitutions at Pro-106 have been observed in isolation; however, the Thr-102-Ile substitution has only been observed in combination with Pro-106-Ser. Here, two new combinations of amino acid substitutions, including a triple-substitution event, are reviewed.

The double-substitution Thr-102-Ile + Pro-106-Thr, termed TIPT, was first observed in *Bidens subalternanas* in Paraguay [67]. The Pro-106-Thr substitution has been reported to confer low-level resistance, and the Thr-102-Ile has been reported to confer resistance only when in combination with Pro-106-Ser [65]. Dose–response conducted between the putative resistant accession and a geographically proximal sensitive accession identified the resistance response. Shikimate accumulation assay results suggest that the resistant accession has an insensitive target-site. No significant variation for non-target-site mechanisms, such as reduced absorption and translocation, metabolism, gene amplification, and vacuolar sequestration, was observed. Gene sequencing revealed the presence of the TIPT substitutions in the resistant accession. No segregation or inheritance studies were conducted. Patent literature describing the TIPT double-substitution was previously reviewed by Sammons and Gaines [64].

The triple-substitution Thr-102-Ile + Ala-103-Val + Pro-106-Ser, termed TAP-IVS, was first identified in *A. hybridus* in Argentina [68]. The effect of substitutions at the Ala-103 position are uncharacterized. Dose–response against a geographically proximal sensitive population identified the resistance response. Shikimate accumulation assay suggests that the resistant population has an insensitive target site. Gene sequencing identified the TAP-IVS triple substitution. While gene amplification was observed within the resistant accession, mRNA levels of EPSP synthase did not correlate with resistance. Structural modelling suggests that TAP-IVS results in a rearrangement of the glyphosate binding domain, and a reduction in interaction sites is observed when compared to wild-type and other resistance-endowing substitutions. Co-segregation and inheritance of the TAP-IVS was not conducted. The impact of Ala-103-Val alone, or in other combinations of substitutions, was not characterized. Functional validation of the TAP-IVS substitutions remains necessary.

### 2.7. Glutamine Synthetase Inhibitors: Group 10 (H)

Resistance to glutamine synthetase inhibitors was last reviewed in 2002 [69]. In short, glutamine synthetase catalyzes the formation of L-glutamine from L-glutamate and ammonia [70]. Two primary isoforms of glutamine synthetase exist in plants: the nuclear-encoded and cytosol-targeted GS1, and the nuclear-encoded and chloroplast-targeted GS2. While inhibition of glutamine synthetase results in an accumulation of ammonium, the primary cause of plant death was thought to occur via inhibition of photorespiration [71]. The transamination of glyoxylate to glycine is a necessary step of photorespiration, where the amino group originates from the fixation of ammonia into glutamine [71,72]. Recently, rapid accumulation of reactive oxygen species has been proposed as the primary reason for glufosinate toxicity [73]. Phosphinotricin (glufosinate) is the only chemistry within Group 10, and appears to inhibit both GS1 [74] and GS2 [75]. The crystal structure of GS1 was developed from *Zea mays* [76] and both GS1 and GS2 from *Medicago truncatula* [77]. 

An Asn-171-Asp substitution was identified in *Lolium perenne* L. spp. *multiflorum* from the US [14]. This is the first report of herbicide resistance due to an altered target site within Group 10. Dose–response revealed resistance when compared to two unrelated sensitive populations. Glutamine synthetase activity assays identified a reduced-sensitivity target site within the resistant accession. Sequence analysis of GS2 identified the Asn-171-Asp substitution. Inheritance and co-segregation analysis were not conducted. Fitness costs associated with Asn-171-Asp were not characterized.

### 2.8. Phytoene Desaturase Inhibitors: Group 12 (F_1_)

Resistance to inhibitors of phytoene desaturase, termed Group 12, was last reviewed in 2014 [78]. Phytoene desaturase mediates the second step of the carotenoid biosynthesis pathway. The enzyme catalyzes the desaturation of 15-cis-phytoene, creating two of the four double bonds required for lycopene synthesis [79]. Phytoene desaturase is a nuclear-encoded, chloroplast-localized enzyme [80]. Herbicides in Group 12 are classified into two classes: pyridazinones and pyridinecarboxamides [57]. In addition, multiple chemistries are unclassified. Recently, the crystal structure of phytoene desaturase alone and in complex with a representative pyridazinone was reported from *Oryza sativa* [79], though no standardized amino acid numbering system appears to be established. The *O. sativa* sequence AAD02489 would make an excellent reference for amino acid numbering. 

As of 2014, evolved target-site resistance was attributed only to substitutions of Arg-304, to either Ser, Cys, or His [78]. Resistance to Group 12 herbicide was only reported within five weed species, including the aquatic weed, *Hydrilla verticillata*. 

A Leu-498-Val, (originally numbered as Leu-526-Val) substitution was first observed in *S. orientale* from Australia [81]. Dose–response revealed a resistant phenotype to a representative pyridinecarboxamide when compared to unrelated sensitive populations. Segregation analysis suggested that the resistant phenotype was mediated by a single, co-dominant to dominant locus. Sequence analysis identified the Leu-498-Val substitution, which is equivalent to the Leu-538 position in *O. sativa*. The Leu-538 position in *O. sativa* corresponds to the causative mechanism of resistance to pyridazinones in numerous species [79]. No fitness costs associated with the Leu-498-Val substitution were observed in the F2 generation when grown in monoculture or in competition with wheat [82]. 

A double-substitution Glu-425-Asp + Leu-498-Val was first observed in *S. orientale* from Australia [83]. Dose–response revealed a resistant phenotype to diflufenican and picolinafen, which are both pyridinecarboxamides. The single-substitution Leu-498-Val was documented as highly resistant to diflufenican, but not picolinafen. Sequence analysis identified the presence of the Glu-425-Asp + Leu-498-Val double substitution. Segregation analysis suggests the observed resistance is mediated by a single, codominant to dominant locus. No fitness costs associated with double substitution were observed in the F2 generation when grown in monoculture or in competition with wheat [82]. Functional validation of the Glu-425-Asp remains necessary.

### 2.9. Protoporphyrinogen Oxidase Inhibitors: Group 14 (E)

Resistance to Group 14 was last reviewed in 2014 [78] and 2018 [84]. Protoporphyrinogen oxidase (PPO) catalyzes the oxidation of protoporphyrinogen IX to protoporphyrin IX [78]. The enzyme is nuclear encoded with two main isoforms: PPO1, which is largely localized to the chloroplast, and PPO2, which is largely localized to the mitochondria [84]. Inhibition of PPO results in an accumulation of protoporphyrin IX within the cytoplasm as a result of protoporphyrinogen IX ‘leaking’ out of organelles. Inhibitors of PPO, termed Group 14, are organized into numerous classes: diphenyl ethers, N-phenylphthalimides, oxadiazoles, oxazolidinediones, phenylpyrazoles, pyridinediones, thiadiazoles, triazinones, and triazolinones [57]. Several chemistries remain unclassified. The crystal structure of PPO2 is characterized from *Nicotiana tabacum* in complex with a phenylpyrazole chemistry [85]. 

To date, all the evolved substitutions reported in peer-reviewed literature for resistance to PPO inhibitors are in PPO2, perhaps because this isoform is dual targeted to both organelles in at least some species [84]. Because PPO inhibitors possess target sites in both mitochondria and chloroplasts, resistance gained at both localizations (e.g., by a single mutation in the gene encoding PPO2) may be required for a resistant phenotype. Resistance-conferring mutations, reported as of 2018, include a deletion of a Gly codon at postion 210, and substitutions of Arg-128 (or 98, depending on the numbering system) to Leu, Gly, or Met. The amino acid numbering system has not been consistent. For instance, Arg-128 describes the position reflective of the *N. tabacum* crystal structure, while Arg-98 describes the position reflective of the *Ambrosia artemisiifolia* enzyme, in which the substitution was first identified. We recommend that the naming convention follow the *N. tabacum* sequence because of the available crystal structure. 

A Gly-399-Ala substitution was first reported in *A. palmeri* from Arkansas, US [86]. Dose–response revealed a resistant phenotype against a representative diphenyl ether chemistry when compared to an unrelated sensitive population. F1 inheritance suggests that the resistant phenotype is dominant. Sequence analysis revealed the Gly-399-Ala substitution. In silico modeling with the available crystal structure suggests that the Gly-399-Ala substitution decreases the binding-pocket size. In vitro PPO enzyme activity assays suggest resistance to representative diphenyl ether, pyrimidinedione, triazolinone, N-phenylphthalimide, phenylpyrazole, thiadiazole, and oxadiazole chemistries and pyraclonil. However, the enzyme containing the Gly-399-Ala had notably reduced enzyme activity in the absence of inhibitors and, therefore, the concentration of the Gly-399-Ala was increased relative to wild-type. As enzyme concentrations were not constant between the Gly-399-Ala and wild-type, further validation in vivo is necessary. 

An Arg-128-Ile substitution was first reported in *Amaranthus tuberculatus* in the US [87]. Substitutions at the Arg-128 position have been reported to confer resistance to diphenyl ether, pyrimidinedione, and triazolinones [88]. Resistant plants from a population survey were identified through delimiting rate screening. DNA of each population was bulked and the gene encoding PPO2, termed *PPX2*, was amplified, barcoded, and subjected to next-generation sequencing. Single-nucleotide polymorphism (SNP) calling revealed the Arg-128-Ile substitution, which was supported by single plant sequence analysis. Pseudo-in vivo enzyme assays using the BT3 hemG system suggest that the Arg-128-Ile substitution confers resistance to a representative diphenyl ether chemistry.

## 3. Discussion

Prior to this review, causal variants for herbicide resistance have largely been considered to be the result of a single nucleotide change from wild-type. With the exception of the TIPS double-substitution in EPSP synthase of *E. indica* [89], which was reported in 2015, nearly all characterized herbicide resistance mechanisms have been explained through a single modification. However, of the 19 target-site mechanisms under review, six cases spanning four herbicide groups require more than one modification from wild-type. The question of which substitutions at a given amino acid position can mediate a resistant phenotype is highly pertinent for resistance management. Previously, this question has been focused to the subset of amino acids which may result from single-nucleotide substitution, as in the case of ALS- and PPO-inhibitors [27,87]. Perhaps in response to the increase in these ‘multiple-modification’ mechanisms, the idea of screening all amino acid substitutions may gain traction.

A primary goal driving the need to characterize herbicide resistance mechanisms is the management of herbicide-resistant weeds. Successful management of herbicide resistance is largely dependent on (a) to which chemistries resistance is conferred, (b) the distribution of the resistance mechanism, and, if present, (c) the associated fitness cost. 

### 3.1. Cross-Resistance Patterns

The exhaustive characterization of cross-resistance patterns associated with a given resistance mechanism is a daunting challenge. For instance, there are 32 published, unique amino acid substitutions reported to confer resistance to at least one Group 2 chemistry. Fifty-seven chemistries within Group 2 have been reported [57], resulting in over 1800 potential unique interactions. While not universal, the use of in vitro enzyme activity assays provides rapid and excellent support for the characterization of herbicide resistance. Furthermore, the high-throughput nature of in vitro assays allows for rapid screening of both multiple herbicide chemical families and multiple members of each chemical family. Together with in silico crystal structure predictions, in vitro assays could be of great use for the development of new herbicidal compounds within existing families. For instance, the observation that the Ala-205-Phe mediates cross-resistance to all tested chemistries, with the exception of florasulam, in vitro could inform the production of new active compounds. 

Translational issues, from in vitro to in planta, have been reported [17]. While having a much lower throughput, functional validation in planta is the gold standard for demonstrating resistance and susceptibility. A uniform and replicable system for in planta functional validation is necessary to facilitate high-throughput screening initiatives. The easily transformable model organism *A. thaliana* is compatible with numerous herbicide groups [15,90]. For herbicide groups which do not provide control of *A. thaliana*, such as ACCase-inhibitors, *S. viridis*, a model system used for the study of millets [91], may be an excellent target. *S. viridis* is a grassy weed readily controlled by numerous Group 1 herbicides [92]. In addition, transformation systems within the species are well characterized and utilize the simple floral-dip technique [93]. While transformation of *S. viridis*, as mediated through floral dip, results in a notably low yield of transformants, floral dip does not require specialized tissue culture capabilities. Should such capabilities be available, *O. sativa* may be another excellent choice [94].

A key problem with the use of transformation-based strategies for the functional validation of herbicide resistance is dosage effects. In this case, dosage effects can be thought of as the increase in protein abundance relative to wild-type. As exemplified by the gene amplification resistance mechanism towards glyphosate, increased expression of a sensitive target protein can result in a resistant phenotype. Even the use of the native promoter can greatly affect herbicide efficacy, as observed by LeClere et al. [15]. Mutagenesis of the target gene would eliminate these concerns. As a particular variant of interest may not be present within mutagenesis collections, a targeted approach is required. 

Fortunately, targeted mutagenesis techniques, such as those mediated through CRISPR/Cas technologies, have become increasingly popular. Through the use of flanking CRIPSR sites, homology-directed repair could be exploited to simply introduce the desired substitutions into the native copy of the target enzyme [95], eliminating dosage-effect concerns. Alternatively, directed base editing through cytosine base editors, which mediate C-to-T nucleotide substitutions, or adenine base editors, which mediate A-to-G substitutions, could avoid the relatively low frequency of homology-directed repair [96]. Through these advances, we believe that the risks involved with in planta functional validation have been greatly diminished and could form the basis for screening resistance-by-chemistry interactions. 

### 3.2. Distribution of Resistance Mechanisms

The distribution of resistance mechanisms is often quantified through routine surveillance across broad geographies. Surveys that monitor herbicide resistance within the same geography over time can provide insights into the effectiveness of management practices in controlling, or encouraging, herbicide resistance. An excellent example is the weed resistance monitoring program in the Northern Great Plains of Canada, which has been routinely conducted since the mid-1990s [97]. While numerous examples of these resistance surveys exist throughout the literature, they are not the primary subject of this review. However, these surveys can be utilized to identify new resistance mechanisms, as illustrated through Nie et al.’s work for the characterization of the Arg-128-Ile, PPO2 substitution of *A. tuberculatus* [87].

### 3.3. Fitness Cost Analysis: A Major Knowledge Gap

Fitness cost analysis has been the subject of past reviews [98,99]. A primary challenge with fitness cost studies is how to control for the genetic background between the R and S plant. From the perspective of this review, of the 19 resistance mechanisms characterized, 13 were compared to unrelated sensitive populations and three to geographically proximal sensitive populations. Dose–responses were not conducted on the remaining three resistance mechanisms. While the generation of nearly-isogenic lines (NILs) or transgenic lines provides the greatest control of genetic background, multiple factors prevent application. NILs are time consuming and impractical to produce in species where backcrosses are challenging. Transgenic methods often require the use of a model system, such as A. thaliana, which abstracts the experiment from a direct field application. Furthermore, issues related to the positional insertion of a given transgene and dosage effects may provide confounding factors, complicating the fitness cost analysis. Finally, the concept that a genetic background itself can compensate for the fitness cost of resistance has support [100]. 

We believe that the use of transgenic methods for fitness cost analysis is undervalued. Targeted mutagenesis techniques, as previously discussed, mitigate many of the systematic errors. Here, we provide some thoughts on the necessity of direct, in-field measurements for fitness cost analysis.

The main goal of a fitness cost analysis is to determine fitness costs associated with a given allele. As fitness cost analysis is resource-intensive, the fitness cost observed is often extrapolated to multiple different species [98] and environments that are subjected to different evolutionary pressures. Therefore, fitness cost analysis within a model system may be as appropriate as such analysis within the native system. As previously discussed, targeted mutagenesis strategies can augment or eliminate issues related to positional and dosage effects. Compensatory effects of the genetic background are poorly understood at a functional level. From a theoretical perspective, Liebig’s law of the minimum [101] may be relevant. Following Liebig’s law, fecundity is determined by the most limiting factor. Within the context of a fitness cost analysis, a cost will only be observed when the given allele results in a greater limitation than observed within the wild-type. Therefore, a genetic background that ‘compensates’ for a resistance trait may be less fit in the greater environment, in the absence of selection, than a genetic background where the fitness cost is observable. An alternative form of compensation would exist within allopolyploids, which can mitigate a present fitness cost of a mutant allele by carrying the wild-type allele within a separate genome. Finally, technical issues, such as positional and dosage effects, can be largely eliminated through targeted mutagenesis, as described previously.

## Figures and Tables

**Table 1:** Identification of mutations conferring target-site resistance to herbicides.

Target Site	Representative Herbicide	Year ^1^
D1 protein	atrazine	1983 [6]
acetolactate synthase	chlorimuron	1992 [8]
tubulin	trifluralin	1998 [9]
acetyl CoA carboxylase	clethodim	2001 [10]
5-enolypyruvylshikimate-3-phosphate synthase	glyphosate	2002 [11]
phytoene desaturase	fluridone	2004 [12]
protoporphyrinogen oxidase	lactofen	2006 [13]
glutamine synthetase	glufosinate	2012 [14]
auxin receptor	2,4-D	2018 [15]

^1^ Indicates first year of publication in peer-reviewed literature of a resistance-conferring mutation in the target-site from a field-evolved weed population.
